# How Does the Seed Pre-Germinative Metabolism Fight Against Imbibition Damage? Emerging Roles of Fatty Acid Cohort and Antioxidant Defence

**DOI:** 10.3389/fpls.2019.01505

**Published:** 2019-11-21

**Authors:** Enrico Doria, Andrea Pagano, Carla Ferreri, Anna Vita Larocca, Anca Macovei, Susana de Sousa Araújo, Alma Balestrazzi

**Affiliations:** ^1^Department of Biology and Biotechnology “L. Spallanzani,” Pavia, Italy; ^2^Consiglio Nazionale delle Ricerche, Research Area of Bologna, Bologna, Italy; ^3^Lipinutragen srl, Laboratorio di Lipidomica, Bologna, Italy; ^4^Instituto de Tecnologia Química e Biológica António Xavier, Universidade Nova de Lisboa (ITQB-NOVA), Oeiras, Portugal

**Keywords:** pre-germinative metabolism, imbibition damage, lipidomics, antioxidant response, *Medicago truncatula*

## Abstract

During seed imbibition, lipids are engaged in membrane reorganization while facing free radical-mediated oxidative injury. In the present work, we explored changes in lipid components at different timepoints of imbibition (0.5, 2, 4, 6, and 8 h) in the legume *Medicago truncatula*, by combining biochemical approaches with targeted lipidomics and untargeted metabolomics. ROS and RNS (reactive oxygen and nitrogen species) accumulation was observed throughout the tested timepoints whereas lipid peroxidation increased at 4 h of imbibition. The seed response to oxidative damage was evidenced by a significant increase in tocopherols starting from 0.5 h of imbibition as well as by the reduction in total thiol content occurring at 2 h of imbibition. Since under physiological conditions, the proper functions of the cell membranes are strongly dependent on the qualitative and quantitative balance of fatty acid residues in phospholipids, the investigation was expanded to the fatty acid cohort of *M. truncatula* seeds. Total saturated fatty acids (SFAs), monounsaturated fatty acids (MUFAs), polyunsaturated fatty acids (PUFAs), omega(ω)-3 and omega(ω)-6 fatty acids showed fluctuations during seed imbibition. The most remarkable finding was the profile of the ω-3 PUFA docosopentaenoic acid (DPA, 7 *cis*, 10 *cis*, 13 *cis*, 16 *cis*, and 19 *cis*-22:5) that showed a peak (up to 1.0% of the total fatty acid content) at 0.5 and 8 h of imbibition, concomitant with the peaks observed in tocopherol levels. It is possible that the observed changes in DPA alter the physical properties of membranes, as reported in animal cells, triggering signaling pathways relevant for the cell defense against oxidative injury. Furthermore, the content and balance between tocopherols and PUFAs is regarded as a determinant of storage stability. No enhancement in *trans*-fatty acids occurred throughout imbibition, suggesting for a proper antioxidant response carried by the seed. Fatty acids profiles were integrated with data from untargeted metabolomics showing changes in lipid sub-pathways, among which fatty acid amide, lyso-phospholipids, and phospholipid metabolism. The emerging lipid profiles and dynamics are discussed in view of the overall imbibition damage generated during *M. truncatula* seed imbibition.

## Introduction

Seed germination is a crucial event in the plant life cycle and, depending on seed quality, this step can severely constrain crop yield with a negative impact on the food chain. As soon as dry seeds are imbibed, complex events take place with precise temporal dynamics, leading to metabolism resumption (Bewley, 1997). The seed coat is a barrier against oxygen diffusion (Bradford et al., 2008); however the level of oxygen partial pressure (pO_2_) can differentially influence germination rates. Park and Hasestein (2016) investigated the response of *Brassica* seeds under different pO_2_ conditions, showing the high degree of seed adaptability in terms of gene expression and metabolite composition. Upon germination, the progressive depletion of oxygen generates conditions that almost achieve anaerobiosis and fermentation is triggered as the main source of cellular ATP, supporting the reduction of electron transferring compounds, e.g. NAD and NADP, and inevitably leading to ROS (reactive oxygen species) accumulation (Ma et al., 2016). At this stage, mitochondria, peroxisomes and the plasma membrane NADPH oxidases are the main sources of ROS, together with lipid catabolism and lipid β-oxidation in the glyoxysomes (Rajjou et al., 2012). ROS levels must be strictly controlled, by means of antioxidant systems, and maintained within a defined range (the so-called “oxidative window”) to avoid any damage to the embryo that would impair germination (Bailly et al., 2008). When germination starts, nitric oxide (NO) production is induced under oxygen limitation. NO turnover, by means of RNS (reactive nitrogen species) scavenging mechanisms, contributes to maintain a redox balance within the seed (Wulff et al., 2009).

Upon rehydration, membrane reorganization anticipates the other molecular events and features the transition from the so-called hexagonal II phase to a lamellar phase that allows to restore the normal function, preventing leakage of cellular components (Ishibashi et al., 2013; Yu et al., 2015). Membrane structure and domain organization strictly depends on lipid biochemical properties and composition. Indeed, the size of the polar head, compared to the hydrophobic tail, affects the lipid behavior in water. Indeed, lipids with a small polar head are characterized by a large negative curvature that promotes membrane organization in the form of inverted micelles (hexagonal II phase). These domains are mainly required to maintain membrane architecture and, within a certain group of lipids, features as increasing chain length and unsaturation number, facilitating the occurrence of hexagonal II phase. Lipids that own similar cross-section area at the level of the polar head and hydrophobic tail, resembling cylinders, from plan lamellar phases (Jouhet, 2013). Changes in environmental temperature can affect the proper membrane reorganization during imbibition, impairing germination rates. The chilling-imbibitional damage has been investigated by Noblet et al. (2017) using lipidomics in maize seeds, revealing that the ability to germinate under cold stress is associated with phospholipid remodeling. On the other hand, global rise in temperature is a severe threat to crop productivity, causing a heat stress-mediated decline in germination rates (Fahad et al., 2017). Membrane thermal stability combined with an effective antioxidant response, is a main component of cellular tolerance to heat stress (Iqbal et al., 2017).

Cellular lipids, particularly polyunsaturated fatty acids (PUFAs), are ROS-sensitive targets and the accumulation of their peroxidation products represent a major cause of decreased seed quality, in terms of germinability and longevity (Devaiah et al., 2007). Due to the cytotoxic effects of hydrogen peroxide (H_2_O_2_) on PUFAs, early seed imbibition is associated with lipid peroxidation that induce membrane damage and malondialdehyde (MDA) production. Lipid peroxidation causes of the so-called imbibition damage (Kovach and Bradford, 1992) occurring when high rainfall induces rapid water up-take by dry seeds after sowing in the field or during imbibitional chilling (Yu et al., 2015). Imbibition damage is one of the major factors affecting seed quality in grain legumes (Powell, 1998), and it has been reported that predisposition to imbibition damage positively correlates with the presence of unpigmented testae (Powell et al., 1986). Mechanisms underlying the repair of damaged membranes in plants exposed to stress and even during seed imbibition are still poorly known; although, Fan et al. (2017) showed that, in *Arabidopsis*, triacylglycerols can sequestrate toxic lipid intermediates, such as free fatty acids, providing protection against oxidative damage. However, the possible role of triacylglicerides in the seed repair response is still unknown.

Within the complex lipid metabolism, the overall profile of saturated fatty acids (SFAs), monounsaturated fatty acids (MUFAs) and PUFAs reflects the multiple membrane arrangements allowing optimized fluidity and permeability as well as suitable receptor- and channel-mediated functions. Under physiological conditions, the proper functions of the cell membrane are strongly dependent on the qualitative and quantitative balance of fatty acids (Ferreri et al., 2017). Therefore, a thorough study of the fatty acid changes during the early step of seed germination should help understanding whether the seed metabolism is oriented towards the increase in unsaturated moieties that influence membrane properties. In this scenario, the impact of oxidative stress can be also evaluated by monitoring *trans*-fatty acids (TFAs) accumulation. TFAs, stereoisomers of the naturally occurring *cis*-fatty acids, are characterized by a linear configuration favoring intermolecular chain-chain interaction stronger than their natural *cis* counterparts (Chatgilialoglu et al., 2014). The occurrence of *trans*-fatty acid isomerization has been correlated to oxidative stress mechanisms mediated by sulfur-containing peptides and proteins, and the small diﬀusible thiyl radicals (RS•) resulting from the degradation of methionine, hydrogen sulfide (H_2_S) and metal-sulfur clusters (Chatgilialoglu et al., 2012; Chatgilialoglu et al., 2014). Under reductive stress conditions, desulfurization at the protein level generates small sulfur-centered radicals that diffuse into the lipid bilayer causing rapid reactions with the PUFA residues and generation of TFAs can be regarded as putative biomarkers of oxidative stress (Chatgilialoglu et al., 2000; Chatgilialoglu et al., 2012; Chatgilialoglu et al., 2014). Nitrogen dioxide (NO_2_^•^) and H_2_O_2_ radicals can also trigger isomerization; however, NO_2_^•^ seems to be more eﬃcient in polar environments (Tzeng and Hu, 2014). The isomerization catalyzed by thiyl radicals is a very efficient process, extensively investigated through mechanistic and kinetic studies, deriving from damaged sulfur-containing proteins diffused within the cellular environment. This event results in a further wave of changes by causing *cis*-to-*trans* isomerization of membrane lipids. However, at the moment, it is not yet well understood whether the process of *cis*-to-*trans* isomerization of membrane lipids represents exclusively a form of cellular damage or it eventually acts as a signal leading to the activation of endogenous defense systems (Chatgilialoglu et al., 2012; Chatgilialoglu et al., 2014; Ferreri et al., 2017). In the context of seed germination, the occurrence and role of TFAs are still poorly explored.

All these issues deserve further investigation to expand the current knowledge on the seed lipid metabolism and to possibly disclose novel hallmarks of seed vigor and/or seed stress. In the present work, we explored changes in lipid metabolism concomitant with early seed imbibition in the model legume *Medicago truncatula* by combining approaches that highlight lipid oxidative damage profiles, as well as the antioxidant response, with targeted fatty acid lipidomics and untargeted metabolomics.

## Materials and Methods

### Plant Materials

For imbibition experiments, *M. truncatula* seeds (Jemalong genotype, kindly provided by Dr. Ana Barradas, Fertiprado L.d.a., Vaiamonte-Monforte, Portugal) were transferred to Petri dishes (diameter 90 mm) containing two filter papers moistened with 2.5 ml H_2_O and kept in a growth chamber at 22°C under light conditions with photon flux density of 150 µmol m^−2^ s^−1^, photoperiod of 16/8 h and 70–80% relative humidity. Seeds with protrusion of the primary radicle were considered germinated and counted seven days after imbibition. Two independent experiments were carried, and for each experiment three replicates (20 seeds per replicate) were analyzed. *M. truncatula* seeds were collected at the indicated time points (0, 0.5, 2, 4, 6, and 8 h), the fresh weight (FW) was measured, and samples were stored in liquid N_2_ for molecular analyses.

### Detection of Free Radical Species

The fluorogenic dye 2′,7′-dichlorofluorescein diacetate (DCF-DA; Sigma-Aldrich, Milan Italy) was used to quantify the levels of ROS (reactive oxygen species) released from dry and imbibed seeds. Following deacetylation by cellular esterases, the dye is converted to a non-fluorescent molecule which is subsequently oxidized by ROS into the highly fluorescent 2′,7′-dichlorofluorescein (DCF). The latter can be detected by fluorescence spectroscopy with maximum excitation and emission spectra of 495 nm and 529 nm, respectively. The assay was carried as described by Macovei et al. (2016), with the following modifications. *M. truncatula* seeds were collected at the indicated timepoints and dried on filter paper. Intact seeds (three seeds per timepoint; two independent experiments) were incubated under dark conditions for 15 min with 50 µl of 10 µM DCF-DA. Subsequently, a volume of 25 µl was pipetted into a 0.2 ml PCR tube and the emitted fluorescence was measured using the green channel (510 ± 5 nm) of a Rotor-Gene 6000 PCR apparatus (Corbett Robotics, Brisbane, Australia), setting the program for one cycle of 30 s at 25°C. This apparatus registers the emission of fluorescence in a similar manner of a fluorimeter. As negative control, a sample containing only DCF-DA was used to subtract the baseline fluorescence. Relative fluorescence was calculated by normalizing samples to controls and expressed as Relative Fluorescence Units (R.F.U.). Dinitrogen trioxide (N_2_O_3_), the main product resulting from the non-enzymatic oxidation of nitric oxide (Planchet and Kaiser, 2006; Ali et al., 2013) was also quantified in dry and imbibed seeds using the above described protocol, except for the fact that detection was carried with diaminofluorescein diacetate (DAF_2_-DA; Sigma-Aldrich).

### Determination of MDA Levels

Polyunsaturated lipids are susceptible to ROS-mediated oxidative stress that triggers a chain reaction resulting in end products such as MDA used as marker of lipid peroxidation and membrane damage. MDA levels were quantified according to Sari et al. (2012) and Zeb and Ullah, (2016), with the following modifications. Seeds were collected at the indicated timepoints and grinded in a mortar until a powder was obtained. An amount of 200 mg of seed powder from each sample was mixed with 5 ml of a H_2_O: 0.5 M HClO_4_ solution (4:1) with some drops of 2% BHT (butylated hydroxytoluene, Sigma-Aldrich, Milan, Italy) in ethanol, in order to precipitate the proteins. The samples were subsequently centrifuged at 4°C for 10 min, then filtered using Whatman No. 1 paper (Whatman Limited, UK). MDA was determined as a thiobarbituric acid reactive substance (TBARS) following its reaction with thiobarbituric acid (TBA) at high temperature. For each sample, an aliquot of 100 µl was mixed with 100 µl of TBA in 1 ml H_2_O and the mixture was heated in a boiling water bath at 95°C for 60 min. The test tubes were cooled at room temperature and absorbance was measured at 254 nm using an UV–visible spectrophotometer (UV-1800, Shimadzu, U.K.). The standard MDA (Sigma-Aldrich) solution (100 µl, in a range of 0.025–0.1 mg/ml) was added in a 1 ml test tube and mixed with TBA (100 µl) as previously described (**Supplementary Figure S1**). All the analyses were performed in triplicates.

### Determination of Total Thiol Content

Non-protein total thiol content was determined according to Voldrich et al. (1995) with the following modifications. Seeds were collected at the indicated timepoints and grinded in a mortar until a powder was obtained. For each sample, an amount of 200 mg of seed powder was incubated with 5 ml of a solution containing 0.12 M NaF, 60 mM ascorbic acid, 0.3 M acetic acid, 2 mM EDTA. The mixture was homogenized overnight at 4°C to allow the extraction of thiols. Subsequently, 200 µl of extract were added to 1 ml of 100 mM KH_2_PO_4_ (pH 8.0), and 50 µl of 40 mM DNTB (5,5′-dithiobis 2-nitrobenzoic acid; Ellman’s reagent) (Sigma-Aldrich) solubilized in methanol. The Ellman’s reagent reacts with a thiol group leading to the formation of a thiol-TNB adduct and the concomitant release of one equivalent of 5-thio-2-nitrobenzoic acid (TNB). Quantification of the thiols is based on the amount of released TNB measured spectrophotometrically at 412 nm (UV-1800, Shimadzu). A standard curve was obtained using concentrations of cysteine in a range of 0.01–0.1 mg/ml (**Supplementary Figure S2**). All the analyses were performed in triplicates.

### Extraction and Analysis of Tocopherols

The extraction procedure was performed as described by Kurilich and Juvic (1999) and Doria et al. (2009) with the following modifications. Seeds were collected at the indicated timepoint and grinded in a mortar and an amount of 500 mg of seed powder from each sample was added to 5 ml of ethanol containing 0.1% butylated hydroxytoluene (BHT) and the mixture was incubated for 10 min at 85°C. Subsequently samples were subjected to saponification by adding 150 µl of 80% potassium hydroxide (KOH) and incubating for 10 min, vortexing a couple of times during the procedure. After adding 3 ml of H_2_O, the samples were placed in ice bath for 3 min and subsequently 3 ml of pure hexane were added. After shaking for 10 min at 800 rpm and centrifuging at 12,000 rpm, the upper layer was transferred into a separate test tube, and the pellet was re-extracted twice more using 2 ml of hexane. The combined hexane fractions were washed with 3 ml of deionized dH_2_O, vortexed, centrifuged for 10 min, and finally transferred into another test tube. Finally, the collected hexane fractions were dried used a vacuum evaporator and the residue dissolved in 200 µl acetonitrile:methanol:dichloromethane 45:20:35 v/v/v) prior to injection into the HPLC system (Kontron Instrument 420 system) (Kontron Instruments, Munich, Germany) equipped with a C18 column (Zorbax ODS column 250 × 4.6 mm, 5 µm, Agilent Technologies). The isocratic mobile phase consisted of acetonitrile:methanol (60:40 v/v), the flow rate was 1.0 ml/min at room temperature, and absorbance was measured at 220 nm. As standard, γ-tocopherol (Sigma-Aldrich) was used for a calibration curve and identified in the chromatogram (**Supplementary Figure S3**). All the analyses were performed in triplicates.

### Targeted Lipidomics

Lipid extracts obtained from *M. truncatula* seeds (dry seeds, and imbibed seeds collected at the indicated timepoints) were analyzed using thin layer chromatography for lipid class separation as previously described (Sansone et al., 2013), detecting mainly triglycerides with traces of monoglycerides and phospholipids. Fatty acid-containing lipids were transformed to the corresponding FAME (fatty acid methyl esters) by adding a 0.5 M KOH solution in MeOH (0.5 ml), quenching the reaction after 30 min with 0.5 ml brine. FAMEs were extracted with *n*-hexane (Merck, HPLC grade, Milan, Italy) for 3 times (2 ml for each step), dried on anhydrous Na_2_SO_4_, evaporated to dryness and analyzed by gas chromatography (GC) as previously described (Scanferlato et al., 2019). FAMEs were analyzed using a GC apparatus (Agilent 6850, Milan, Italy) equipped with a 60 m × 0.25 mm × 0.25 µm (50%-cyanopropyl)-methylpolysiloxane column (DB23, Agilent, U.S.A.) and a flame ionization detector with the following oven program: temperature started from 165°C, held for 3 min, followed by an increase of 1°C min^−1^ up to 195°C, held for 40 min, followed by a second increase of 10°C min^−1^ up to 240°C, held for 10 min. A constant pressure mode (29 psi) was chosen with helium as carrier gas. Methyl esters were identified by comparison with the retention times of authentic samples. All reference fatty acid methyl esters (methyl oleate, methyl elaidate, methyl linoleate, linoleic acid methyl ester isomer mix, linolaidic acid methyl ester, conjugated octadecadienoic acid methyl esters, methyl palmitate, and methyl stearate) were purchased from Sigma-Aldrich.

### Metabolomic Profiling

Non-targeted metabolomic profiling was performed by Metabolon Inc. (Durham, NC, U.S.A.) (www.metabolon.com), as previously reported by Pagano et al. (2019). Global metabolomic profiling was performed on methanol extracts from powdered frozen samples (100 mg) of dry seeds (DS) and seeds imbibed with H_2_O for 2 h (W2), 8 h (W8). Metabolomic analyses were conducted in biological triplicates. Metabolites were identified by automated comparison of the ion features of the experimental samples with a reference library of chemical standard entries that included retention time, molecular mass calculated from measured *m/z* ration, preferred adducts, and in-source fragments as well as associated MS/MS spectra and curated by visual inspection for quality control using a in house resources (Evans et al., 2009; Oliver et al., 2011). Raw area counts for each biochemical compound were normalized by dividing each sample value per sample fresh weight. Then, this value was rescaled by dividing each sample value by the median value for the specific biochemical. For missing data (nulls), the minimum observed values for that compound was imputed. Prior to data analysis, scaled imputed data was log transformed. Metabolites were also mapped onto general biochemical pathways, as provided in the Kyoto Encyclopedia of Genes and Genomes (KEGG) (www.genome.jp/kegg/) and Plant Metabolic Network (PMN) (www.plantcyc.org/). Welch’s two-sample *t*-tests were used to determine whether or not each metabolite had significantly change in abundance, focusing on comparisons between samples of different timepoints (W2 *versus* DS; W8 *versus* W2). Prior to statistical computations, scaled imputed data was log transformed (natural logarithm). An estimate of false discovery rate for the list of all identified compounds was also provided, taking into account the multiple comparison tests conducted in metabolomic-based studies (Storey and Tibshirani, 2003). Metabolites that achieved statistical significance (*P* ≤ 0.05 and *q* ≤ 0.1) were considered differentially accumulated (DA) and selected for further analysis.

### Statistical Analysis

Principal component analysis (PCA) was conducted using the software resources available at Metaboanalyst 4.0 (www.metaboanalyst.ca) following user’s guide specifications (Xia and Wishart, 2016). Two sets of PCA were carried out using different variables: (1) in the first one, ROS, RNS, MDA, thiols, tocopherols and fatty acid cohorts (SFA, MUFA, FUPA, ω-6, ω-3, and trans-fatty acids) were considered, (2) while in the second one, the individual categories of lipids (myristic acid, palmitic acid, stearic acid, arachidic acid, behenic acid, linolelaidic acid, linoleic acid, γ-linolenic acid, dihomo γ-linolenic acid, α-linolenic acid, sapienic acid, palmitoleic acid, oleic acid, vaccenic acid, docosopentaenoic acid, and docosohexaenoic acid) were taken into consideration to evaluate the influence of each component.

As for biochemical and lipidomic data, for each treatment, two independent experiments were performed, using three replicates. Asterisks indicate statistically significant differences compared to control (dry seeds, DS) determined using Student’s *t*-test (*, *P* < 0.01).

## Results

### Water Up-Take During *M. truncatula* Seed Imbibition Results in Enhanced Production of ROS and RNS

**Figure 1:**
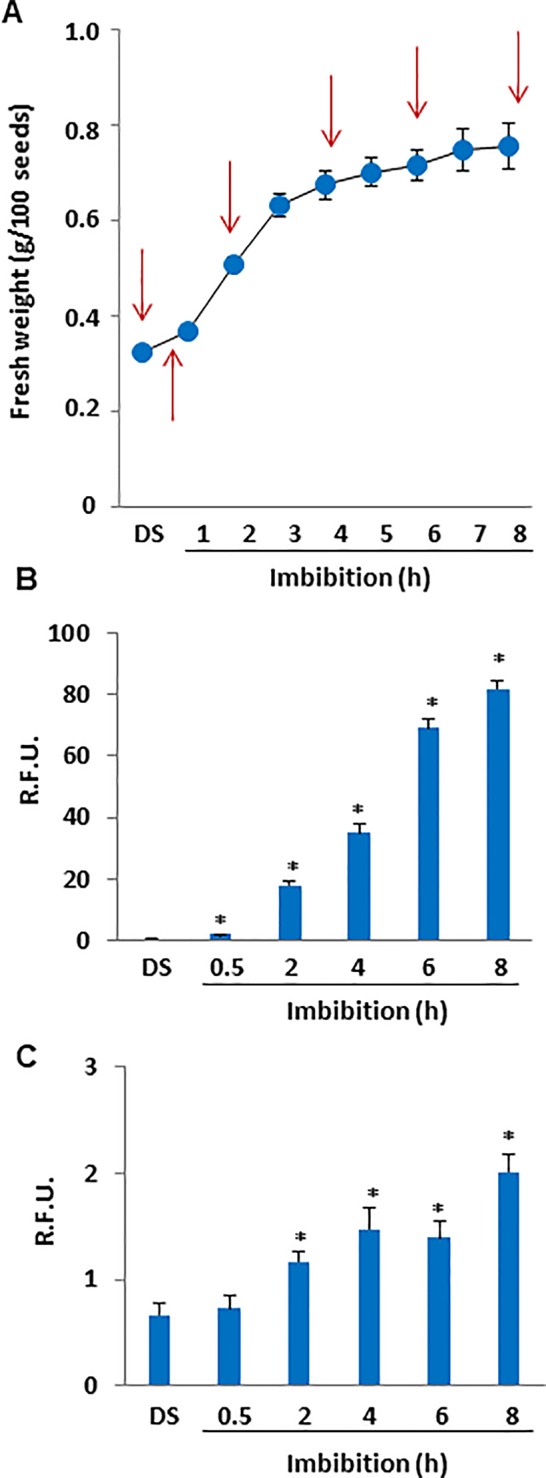
**(A)** Imbibition curve of *M. truncatula* seeds. Arrows indicate the selected time points (0, 0.5, 2, 4, 6, and 8 h) for analyses. **(B)** ROS levels measured in dry and imbibed seeds using the DCF-DA fluorescent dye. **(C)** N_2_O_3_ levels measured in dry and imbibed seeds using the DAF_2_-DA fluorescent dye. Values are expressed as mean ± SD of three independent replicates with 20 seeds for each replicate. Asterisks indicate statistically significant differences determined using Student’s *t*-test (*P* < 0.01). N_2_O_3_, dinitrogen trioxide; R.F.U., relative fluorescence unit; ROS, reactive oxygen species; DCF-DA, dye 2′,7′-dichlorofluorescein diacetate; DAF_2_-DA, diaminofluorescein diacetate.

The gain in seed fresh weight occurring during imbibition was measured to monitor water up-take (**Figure 1A**). Phase I of imbibition (from 0 to 8 h) was characterized by a rapid and significant (*P* = 0.000748) increase in fresh weight, from 0.3227 ± 0.0010 g per 100 seeds to 0.7378 ± 0.0200 g per 100 seeds. Subsequently, *M. truncatula* seeds were collected at selected timepoints during imbibition and ROS levels were measured in dry seeds (DS) and rehydrated seeds (0.5, 2, 4, 6, and 8 h of imbibition) using the DCF-DA fluorescent dye (**Figure 1B**). The fluorescence measured throughout the tested timepoints revealed a significant increase in ROS levels starting from 2 h of imbibition (2.03 ± 0.04 R.F.U., up to 25-fold, compared to DS), and subsequently a progressive ROS accumulation was observed until the end of the experiment (8 h, (81.9 ± 2.51 R.F.U., estimated increase up to 115-fold) (**Figure 1B**). The DAF_2_-DA fluorescent dye was used to detect dinitrogen trioxide (N_2_O_3_), the main product of the non-enzymatic oxidation of NO (**Figure 1C**). In our investigation, the fluorescence measured throughout the tested timepoints revealed no significant changes after 30 min of imbibition compared to DS whereas significant increases in N_2_O_3_ levels were observed at the subsequent timepoints of imbibition. After 8 h, the estimated increase in N_2_O_3_ was 3-fold (2.01 ± 0.16 R.F.U), compared to DS (0.66 ± 0.11 R.F.U).

### Lipid Peroxidation and Changes in Total Thiol and Tocopherol Contents Are Observed During *M. truncatula* Seed Imbibition

**Figure 2:**
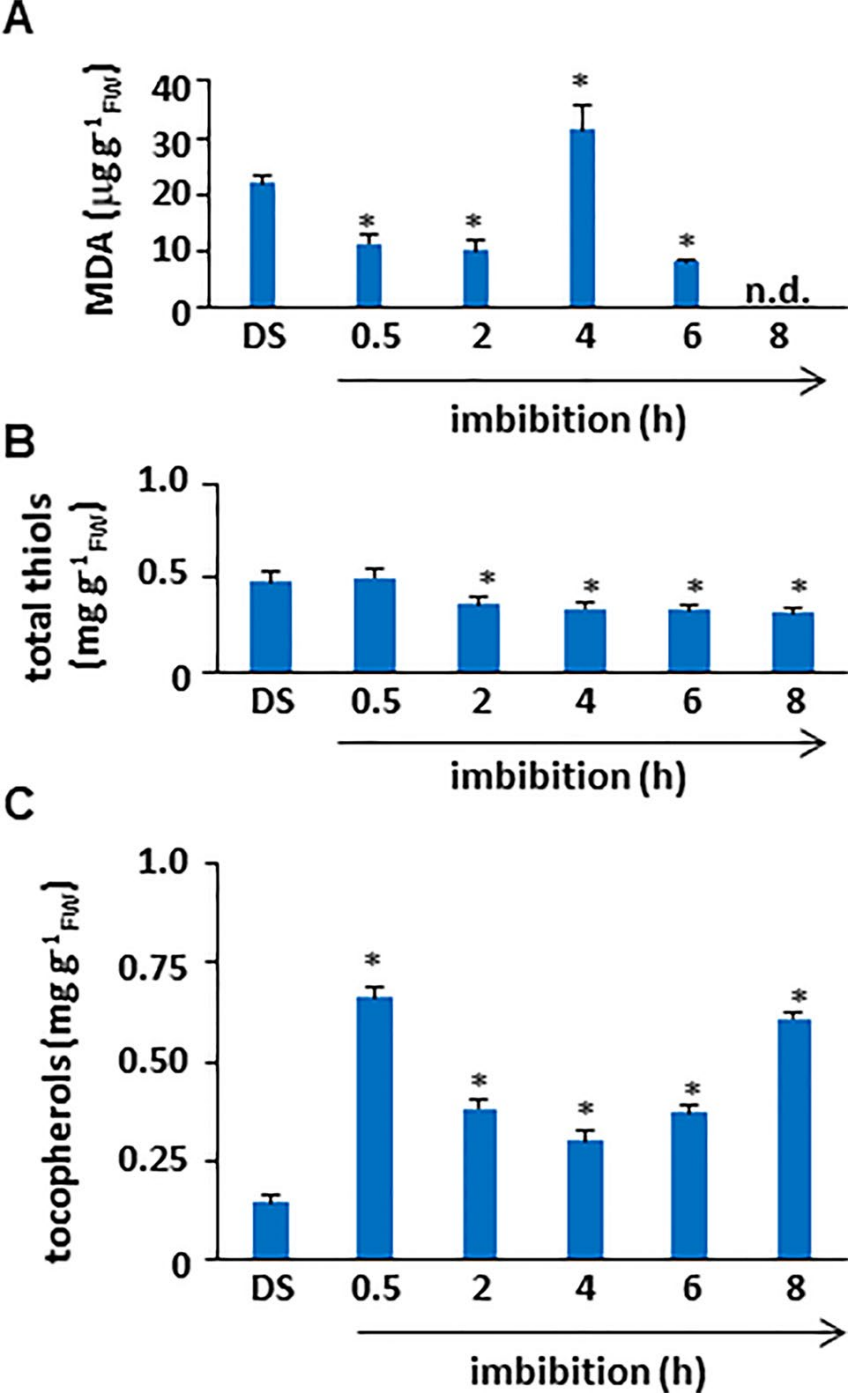
**(A)** Quantification of lipid peroxidation levels in *M. truncatula* dry and imbibed seeds. **(B)** Total thiol content in *M. truncatula* dry and imbibed seeds. **(C)** Total tocopherol content in *M. truncatula* dry and imbibed seeds. Values are expressed as mean ± SD of three independent replicates with 20 seeds for each replicate. Asterisks indicate statistically significant differences determined using Student’s *t*-test (*P* < 0.01). MDA, malondialdehyde; FW, fresh weight.

To assess the impact of ROS accumulation on lipid membrane integrity during imbibition, lipid peroxidation profiles of *M. truncatula* seeds were evaluated in terms of MDA levels (**Figure 2A**). The estimated amount of MDA in the dry seed was 21.91±1.4 µg/g_FW_. After 30 min of imbibition, a significant decrease in MDA levels (11.48±1.62 µg/g_FW_) occurred, similarly to what observed subsequently at 2 h (10.25±1.62 µg/g_FW_). A peak in lipid peroxidation (MDA concentration of 31.27±4.53 µg/g_FW_) was detected at 4 h of imbibition, followed by a decrease (MDA concentration of 8.31±0.31 µg/g_FW_) at 6 h. At the end of the experiment no detectable levels of MDA were reported. As shown in **Figure 2B**, the estimated total thiol content in *M. truncatula* dry seeds was 0.48 ± 0.05 mg/g_FW_. At the beginning of imbibition (0.5 h), no significant changes occurred (0.50 ± 0.05 mg/g_FW_). Subsequently, a significant decrease was observed at 2 h (0.36 ± 0.03 mg/g_FW_). The same levels of total thiols were maintained throughout the subsequent timepoints until the end of the experiment (8 h, 0.315 ± 0.03 mg/g_FW_). The reported decrease in thiol levels is indicative of enhanced oxidative stress causing an imbalance in the cellular redox state and correlates with the observed increase in lipid peroxidation. As shown in **Figure 2C**, the estimated tocopherols content in *M. truncatula* dry seeds was 0.15 ± 0.01 mg/g_FW_. At the beginning of imbibition, a significant increase was observed (0.66 ± 0.02 mg/g_FW_). Subsequently, a decrease was observed at 2 h (0.38 ± 0.02 mg/g_FW_) when compared with the levels observed at 0.5 h; however, the thiol levels at 2 h were significantly higher than those in DS. A similar pattern was detected also at 4 h and 6 h (0.30 ± 0.02 and 0.37 ± 0.01 mg/g_FW_, respectively). Further accumulation occurred at the end of the experiment and (8 h, 0.61 ± 0.01 mg/g_FW_). Accumulation of tocopherols at 30 min from the beginning of water up-take suggests that the seed antioxidant response has already started.

### Targeted Fatty Acids Profiles in Dry and Imbibed *M. truncatula* Seeds: The SFA, MUFA and PUFA Cohorts

**Figure 3:**
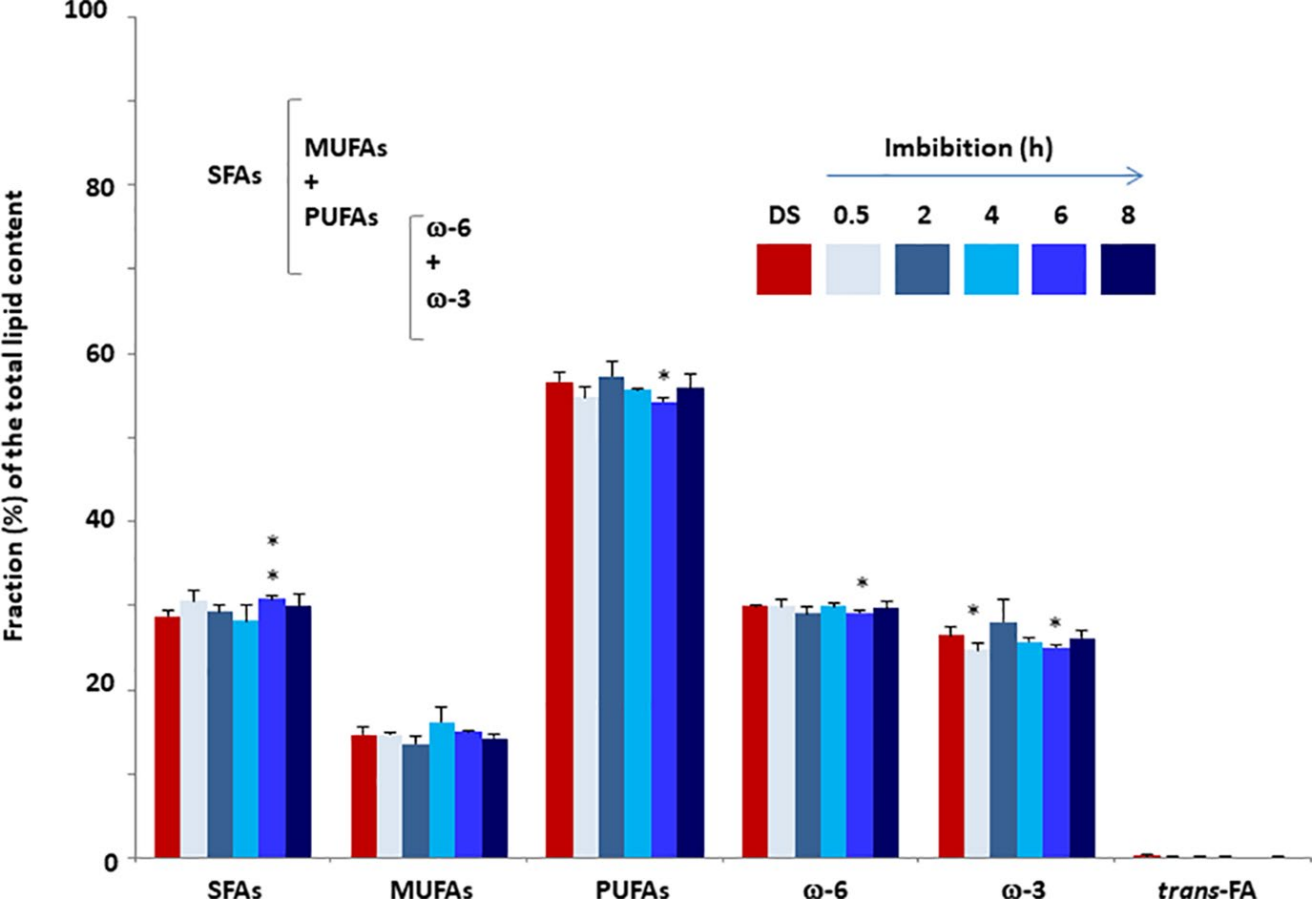
Fatty acid content of *M. truncatula* seeds measured in dry seeds (DS) and imbibed seeds at different time points during rehydration. Values are expressed as mean ± SD of three independent replicates with 20 seeds for each replicate. Asterisks indicate statistically significant differences determined using Student’s *t*-test (*P* < 0.01). SFAs, total saturated fatty acids; MFAs, monounsaturated fatty acids; PUFAs, polyunsaturated fatty acids; ω-3, omega-3 fatty acids; ω-6, omega-6 fatty acids.

FA quantification was performed *M. truncatula* seeds collected at different time points (dry seed, 0.5, 2, 4, 6, and 8 h) during imbibition. The estimated values of total SFAs, MUFAs, PUFAs, omega-3 fatty acids (ω-3), and omega-6 fatty acids (ω-6) are expressed as relative percentage (%rel) of the total FA content identified with appropriate standards (**Figure 3**). The three families of fatty acids, SFA, MUFA, and PUFA, were investigated using the main representative components. As shown in **Figure 3**, the total SFAs in dry seeds accounted for 28.76±0.57% of the total FAs. During imbibition, a significant increase (up to 30.73 ± 0.40%) was observed only at 6 h. The SFA cohort included myristic (14:0), palmitic (16:0), stearic (18:0), arachidic (20:0), and behenic (22:0) acids. The levels of these representative fatty acids observed in dry and imbibed *M. truncatula* seeds are shown in **Table 1** as % of the total FA content, recognized by appropriate standards. Palmitic acid was the most prominent component (21–22%) whereas myristic acid was barely detected (<1%). As shown in **Table 1**, a significant increase in myristic acid and stearic acid contents was observed at 6 h of imbibition, compared to dry seeds. Similarly, a significant increase in behenic acid content was observed at 4 h of imbibition, compared to dry seeds.

**Table 1:** The SFA (saturated fatty acid) cohort in dry and imbibed *M. truncatula* seeds. For each representative component, concentration is expressed as percentage (%) of the total fatty acid content, recognized by appropriate standards.

Treament	Myristic acid (14:0)	Palmitic acid (16:0)	Stearic acid (18:0)	Arachidic acid (20:0)	Behenic acid (22:0)
DS	0.47± 0.02	21.47 ± 0.48	3.19 ± 0.11	2.03 ± 0.14	1.66 ± 0.08
*Imbibition*					
0.5 h	0.60 ± 0.14	22.60 ± 1.03	3.50 ± 0.18	2.09 ± 0.13	1.80 ± 0.21
2 h	0.94 ± 0.74	21.33 ± 1.22	3.11 ± 0.37	2.12 ± 0.10	1.69 ± 0.15
4 h	0.26 ± 0.24	21.56 ± 0.86	2.30 ± 2.00	2.22 ± 0.12	1.87 ± 0.10*
6 h	0.49 ± 0.03*	22.72 ± 0.35	3.53 ± 0.10*	2.22 ± 0.24	1.76 ± 0.15
8 h	0.45 ± 0.12	22.63 ± 0.60	3.20 ± 0.38	2.05 ± 0.10	1.57 ± 0.27

Values are the mean ± SD of three independent replicates with 20 seeds for each replicate. Asterisks indicate statistically significant differences determined using Student’s t-test (*, P < 0.01). DS, dry seed.

No significant changes occurred in the total MUFAs content when comparing the dry and imbibed seeds. The estimated amount of MUFAs was in the range 13–17% of the total FAs. The MUFA cohort hereby analyzed includes palmitoleic acid (9 *cis*-16:1) and palmitelaidic acid (9 *trans*-16:1), sapienic acid (6 *cis*-16:1), oleic acid (9 *cis*- and 9-*trans* 18:1 isomers), *cis*-vaccenic acid (11 *cis*-18:1). The levels of these representative FAs observed in dry and imbibed *M. truncatula* seeds are shown in **Table 2** as % of the total FA content, recognized by appropriate standards. Oleic acid was the most prominent component (12–14% of the total FAs) among MUFAs. The *trans* isomers of MUFAs were not detected.

**Table 2:** The MUFA (monounsaturated fatty acid) cohort in dry and imbibed *M. truncatula* seeds. For each representative component, concentration is expressed as percentage (%) of the total fatty acid content, recognized by appropriate standards.

Treament	Palmitelaidic acid (9 *trans* - 16:1)	Sapienic acid (6 *cis* - 16:1)	Palmitoleic acid (9 *cis* - 16:1)	Elaidic acid (9 *trans* - 18:1)	Elaidic acid (9 *cis* - 18:1)	Vaccenic acid (11 *cis* - 18:1)
DS	n.d.	0.11 ± 0.03	0.15 ± 0.09	n.d.	13.70 ± 0.86	0.68 ± 0.05
*Imbibition*						
0.5 h	n.d.	0.15 ± 0.06	0.14 ± 0.05	n.d.	13.71 ± 0.33	0.70 ± 0.04
2 h	n.d.	0.11 ± 0.05	0.11 ± 0.04	n.d.	12.67 ± 0.93	0.67 ± 0.05
4 h	n.d.	0.14 ± 0.03	1.20 ± 1.80	n.d.	14.00 ± 0.76	0.72 ± 0.12
6 h	n.d.	0.15 ± 0.06	0.12 ± 0.03	n.d.	14.10 ± 0.18	0.68 ± 0.11
8 h	n.d.	0.24 ± 0.15	0.21 ± 0.10	n.d.	13.00 ± 0.29	0.75 ± 0.06

Values are the mean ± SD of three independent replicates with 20 seeds for each replicate; n.d., not detected.

In the case of PUFAs, the estimated level in dry seed was 56.60±1.21% and the only significant change was the decrease detected at 6 h of imbibition (54.21±0.48%). The PUFA cohort includes linoleic acid (18:2, ω-6), γ-linolenic acid (18:3, ω-6), dihomo γ-linolenic acid (20:3, ω-6), α-linolenic acid (18:3, ω-3), docosopentaenoic acid (7 *cis*, 10 *cis*, 13 *cis*, 16 *cis*, 19 *cis*-22:5, ω-3, DPA) and docosahexaenoic acid (4 *cis*, 7 *cis*, 10 *cis*, 13 *cis*, 16 *cis*, 19 *cis*-22:6, ω-3, DHA). The levels of these representative FAs observed in dry and imbibed *M. truncatula* seeds are shown in **Table 3** as % of the total FA content, recognized by appropriate standards. Linoleic acid, the parental fatty acid of the ω-6 series, was the most prominent component (28–30%) followed by α-linolenic acid, the parental fatty acid of the ω-3 series (23–27%). All the other representative FAs were detected at very low levels (< 1%). Moreover, a significant decrease in linoleic acid was observed at 6 h of imbibition, compared to dry seeds while the amounts of ω-6 γ-linolenic acid was significantly enhanced at 8 h.

**Table 3:** The PUFA (polyunsaturated fatty acid) cohort in dry and imbibed *M. truncatula* seeds. For each representative component, concentration is expressed as percentage (%) of the total lipid content.

Treament	Linolelaidic acid (*trans* - 18:2)	Linoleic acid (18:2, ω - 6)	γ-Linolenic acid (18:3, ω - 6)	Dihomo γ-linolenic acid (20:3, ω - 6)	α-Linolenic acid (18:3, ω - 3)	Docosahexaenoic acid (22:6, ω - 3)
DS	0.08 ± 0.03	29.67 ± 0.31	0.10 ± 0.01	0.10 ± 0.03	25.80 ± 1.05	0.60 ± 0.01
*Imbibition*						
0.5 h	0.16 ± 0.09	29.53 ± 0.93	0.16 ± 0.07	0.18 ± 0.08	23.08 ± 0.33	0.75 ± 0.13
2 h	0.16 ± 0.10	28.59 ± 0.82	0.19 ± 0.15	0.21 ± 0.06	26.44 ± 3.21	0.87 ± 0.25*
4 h	0.10 ± 0.08	29.58 ± 0.45	0.13 ± 0.06	0.13 ± 0.03	24.38 ± 0.42	0.71 ± 0.14
6 h	0.06 ± 0.01	28.88 ± 0.25**	0.11 ± 0.03	0.10 ± 0.04	23.75 ± 0.50	0.72 ± 0.11
8 h	0.09 ± 0.02	29.43 ± 0.85	0.20 ± 0.05*	0.10 ± 0.06	24.38 ± 1.53	0.67 ± 0.31

Values are the mean ± SD of three independent replications with 20 seeds for each replication. Asterisks indicate statistically significant differences determined using Student’s t-test (* P < 0.05; ** P < 0.01). DS, dry seed. n.d., not detected.

The ω-3 DPA level was the first value shown to significantly and strongly increase at 0.5 h from the beginning of imbibition and, subsequently, the ω-3 DPA content was steadily enhanced until 8 h, reaching a 3-fold higher amount compared to the initial one. The ω-3 and ω-6 PUFAs are relevant structural cell components and, when included into the membrane phospholipids, they can influence membrane fluidity and permeability as well as the activity of membrane-associated enzymes. The observed increase of a ω-3 PUFA in the seed lipid pool at the onset of germination suggests for the occurrence of specific molecular and metabolic events required to sustain this growth process. The level of total PUFAs remained approximately the same throughout the imbibition.

The *trans*-fatty acids were barely detected (< 0.4%) in both dry and imbibed seeds, and the finding that no enhancement occurred confirms that the oxidative/free radical-mediated processes associated with imbibition were properly controlled by the seed antioxidant machinery.

### Non-Targeted Lipidomics Provides an Additional Snapshot of the Seed Lipid Metabolism

In order to gather additional information on the overall changes in lipid metabolism occurring during seed imbibition, non-targeted metabolomics data related to the lipid super-pathway and sub-pathways were retrieved from Pagano et al. (2019) (**Supplementary Table S3**; **Supplementary Material, Data Sheet 2**). Based on the experimental design from this previous work, it was possible to evaluate those compounds belonging to the lipid pathways that were differentially accumulated during *M. truncatula* seed imbibition, at the transition between dry seed (DS) and imbibed seed (2 h) and at the transition between 2 h and 8 h of imbibition (hereby referred to as W2/DS and W8/W2, respectively).

As shown in **Table 4**, at the DS/W2 transition, only five metabolites could meet the significance threshold (*P* < 0.05, *q* < 0.1) imposed. The free FAs oleic acid and vaccenic acid (18:1) were differentially accumulated at the W2/DS transition in *M. truncatula* seeds. Oleic acid is a ω-9 MUFA, stable to oxidation compared to PUFAs, able to enhance the activity of antioxidants as tocopherols as well as to confer protection against lipid peroxidation. The kinked lipid chain of oleic acid favors the organization of cell membranes, helps to maintain the hydration level, increasing membrane fluidity. In bacteria, the presence of vaccenic acid has been associated with mechanisms that confer thermal regulation in response to temperature changes (Mansilla et al., 2004). The fatty acid amides palmitoyl ethanolamide and oleoyl ethanolamide were differentially accumulated at the W2/DS and W8/W2 transitions. Fatty acid amides showing biological activities at very low concentrations that trigger signalling pathways among which those involved in seed germination (Keereetaweep et al., 2013; Keereetaweep et al., 2015). Similarly, the lysophospholipid 1-palmitoyl-glycero-3-phosphocholine (16:0), and the phospholipids glycerol-3-phosphate and glycerophosphoethanolammine showed differential accumulation at the W2/DS transition, possibly suggesting for a role in lipid-mediated signaling. Thus, besides the targeted fatty acid lipidomics, also non-targeted metabolomic analysis provides evidence for changes in lipid dynamics during *M. truncatula* seed imbibition.

**Table 4:** List of metabolites belonging to the lipid super-pathway and relative sub-pathways showing changes (*, *P* ≤ 0.05 and *q* ≤ 0.1) at the transition between dry seed and 2 h of imbibition (W2/DS) and at the transition between 2 h and 8 h of imbibition (W8/W2).

Lipid sub-pathway	Metabolite	W2/DS (Log_2_FC)	W8/W2 (Log_2_FC)
Free fatty acid	Oleic acid (18:1)/vaccenic acid (18:1)	0.65*	1.06
Fatty acid, hydroxy	2-hydroxyglutarate	0.99	0.59*
Fatty acid ester	Palmitoylcholine	0.69	0.48*
Fatty acid amide	Palmitoyl ethanolamide	0.81	0.45*
	Oleoyl ethanolamide	0.62*	0.69*
Lyso-phospholipids	1-plamitoyl-glycero-3-phosphocholine (16:0)	0.27*	1.08
Phospholipid Metabolism	Glycerol-3-phosphate	0.56*	0.97
	Glycerphosphoethanolammine	0.23*	1.0

DS, dry seed; FC, fold change; W, water.

### PCA Analysis Shows a Clear Distinction Between Imbibition Timepoints Due to Oxidative Damage and Fatty Acids Profiles

**Figure 4:**
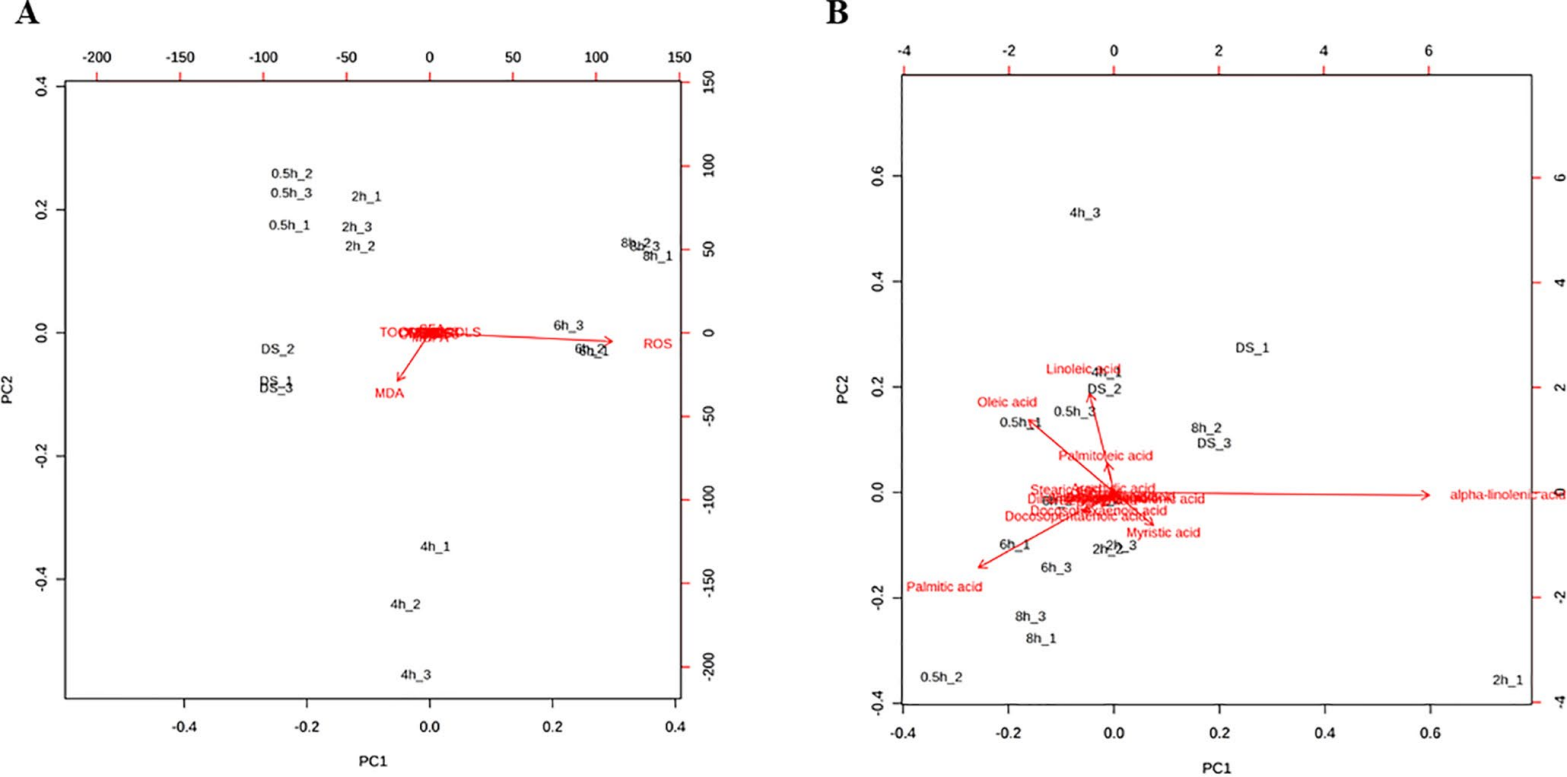
Principal Component Analysis loading plots presenting the variables mostly contributing to the differences observed among samples. **(A)** Position of the variables ROS, RNS, MDA, thiols, tocopherols and fatty acid cohorts (SFA, MUFA, FUPA, ω-6, ω-3, and trans-fatty acids) projected in the plane as determined by the first two principal components. **(B)** Position of the fatty acid variables (myristic acid, palmitic acid, stearic acid, arachidic acid, behenic acid, linolelaidic acid, linoleic acid, γ-linolenic acid, dihomo γ-linolenic acid, α-linolenic acid, sapienic acid, palmitoleic acid, oleic acid, vaccenic acid, docosopentaenoic acid, docosohexaenoic acid) projected in the plane as determined by the first two principal components.

**Figure 5:**
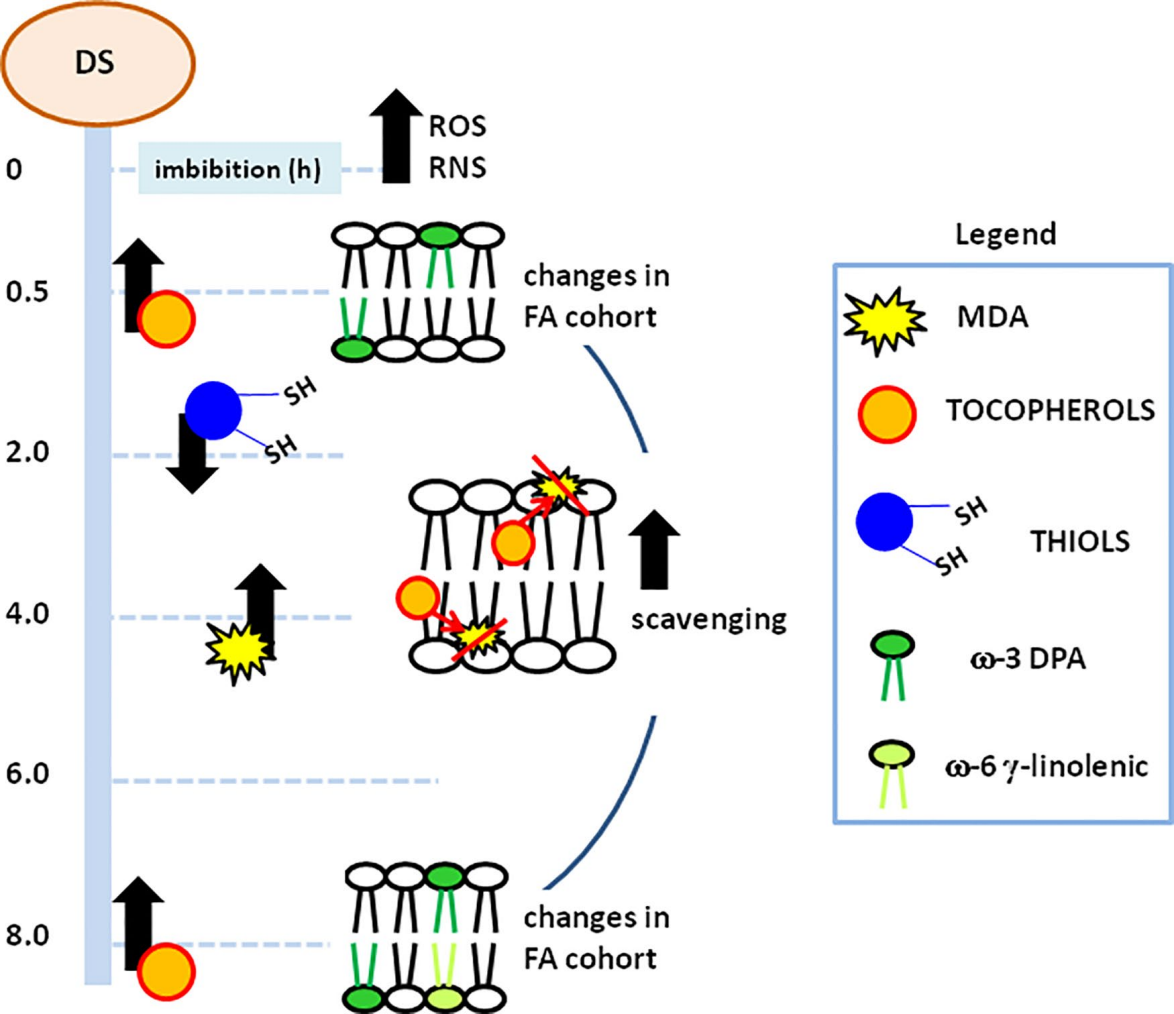
Schematic representation of the metabolic events occurring along the different pathways monitored during *M. truncatula* imbibed seeds. Processes correlated with membrane stability and function and their temporal profiles are highlighted. MDA, malondialdehyde; DPA, docosopentaenoic acid; ROS, reactive oxygen species; RNS, nitrogen reactive species.

PCA was used to explore how the samples differ among them and which variables are mostly responsible for the observed differences. When considering the measured variables ROS, RNS, MDA, thiols, tocopherols and fatty acid cohorts, two principal components (PC1 and PC2) were extracted, accounting for a striking 99.4% of the variance (**Supplementary Figure S4**). Within this variance, PC1 contributed the most (92.9%) whereas PC2 accounted for 6.5% of total variance. The distribution of the samples within the loading plot reveal that each imbibition timepoint were distinctively separated from each other; however, samples DS, 0.5 h, and 2 h were closer to each other, a pattern observed also in the case of the 6 h and 8 h samples, while the 4 h samples were most distant. Subsequently, a biplot representation was analyzed in order to evidence which variables mostly contributed to the sample differentiation. Two biplots were constructed to better differentiate between variables (**Figure 4**). In the first case, when ROS, RNS, MDA, thiols, tocopherols and fatty acid cohorts were considered, it is possible to observe that the main variables are represented by ROS (strongly correlated with PC1, 0.984) and MDA (strongly correlated with PC2, −0.979) (**Figure 5A**, **Supplementary Table S1**). In the second case, when each fatty acid detected were taken into consideration, the main represented variables were α-linoleic acid (correlated with PC1, 0.874) and linoleic acid (correlated with PC2, 0.645) (**Figure 5B**, **Supplementary Table S2**). Other shown variables, although with lower correlation index, include oleic acid (0.473), palmitic acid (−0.491), palmitoleic acid (0.192), and myristic acid (−0.216). This analysis shows that when all the imbibition timepoints and variables measured are taken into consideration, the main differences between samples are given by indicators of oxidative damage (ROS, MDA) and specific fatty acids (linoleic acid, α-linoleic acid) that are affected by these conditions.

## Discussion

This work provides, for the first time, insights into the early events involving cellular lipids during seed imbibition in the legume *M. truncatula*. Imbibition damage results from both the physical and metabolic impact of rapid water up-take by dry seed and lipids are among the macromolecules attacked by ROS. The observed cyclic changes in MDA levels might reflect the biphasic nature of water up-take, as reported by Meyer et al. (2007). The first step of seed imbibition is relatively slow while the second step features rapid water up-take as a result of cotyledon hydration. Both steps are associated with ROS production and related lipid peroxidation. Thus, high lipid peroxidation levels should be expected during the first step, as well as a subsequent transient decline followed by a second peak. The pattern hereby reported for *M. truncatula* has been previously described in soybean seeds by Lisiak et al. (2009). The total thiol content (either in the free form as oxidized or reduced glutathione, or thiols bound to proteins) was also measured in *M. truncatula* seeds, revealing a significant depletion starting from two hours of imbibition with no further significant changes throughout the tested timepoints. Such a profile reflects not only the antioxidant role of thiols but their involvement in the regulation of cellular processes mediated by thiol/disulfide exchange reactions (Kranner and Grill, 1993). Changes in tocopherols levels hereby reported during *M. truncatula* seed imbibition, might be due to their well-known action as scavengers of the lipid peroxidation products. Sattler et al. (2004) demonstrated the key role played by these lipophilic antioxidants in preventing lipid peroxidation during seed germination in *Arabidopsis*. Indeed, *Arabidopsis* mutants lacking tocopherols showed increased lipid peroxidation levels during seed germination and early seedling development, evidencing the biological role of these molecules (Sattler et al., 2004).

The seed repair response at the level of cellular membranes challenged with oxidative stress is a relevant issue that still deserves to be investigated. Lipidomics represents a valuable and informative tool, however, the dynamics of lipid metabolism have been so far investigated mainly in relation to the seed potential as a source of vegetable oil (Zhi et al., 2017; Woolfield et al., 2018) or in transgenic seeds engineered to accumulate ω-3 long chain PUFAs, as DPA and DHA (Zhou et al., 2014). In this context, the present work offers an original contribution, particularly concerning legumes. The FA profiles hereby reported in *M. truncatula* differ from those described in *Medicago sativa* by Huang and Grunwald (1990) who found a decrease in palmitic acid and oleic acid and increased levels of linoleic and linolenic acids. Thus, this reinforces the species-specific differences observed in FA metabolism in seeds.

Looking inside the temporal window of *M. truncatula* seed imbibition, the lipidomic follow-up of fatty acid changes highlighted the highest number of significant variations at 6 h. At this timepoint, both an increase of saturated fatty acids (myristic acid,14:0 and stearic acid, 18:0) and a decrease of ω-6 linoleic acid and ω-3 α-linolenic acid, were observed. Indeed, the decreased FA levels are congruent with the accumulation of peroxidation products.

The FA lipidomics revealed an intriguing feature of *M. truncatula* seeds, that is the ability to accumulate the ω-3 DPA, already visible at 30 min of imbibition. It has been reported that replacement of DHA with DPA alters the physical properties of membranes, influencing the function of integral membrane proteins (Eldho et al., 2003). At the moment, we can only speculate on the biological significance of such a change during seed imbibition. To date, the enhancement of the long-chain ω-3 DPA and DHA levels in a plant system has been achieved only in transgenic *Arabidopsis* plants overexpressing an optimal set of genes coding for Δ6-desaturase, Δ6-elongase, and Δ5-desaturase, the core enzymes in the synthesis of long-chain PUFAs (Ruiz-Lopez et al., 2013). The observed profiles of SFAs, MUFAs and PUFAs reflect the seed ability to withstand oxidative damage from the moment when water up-take starts. Mene-Saffrane et al. (2009) showed that α-linolenic acid (18:3, ω-3), found at high levels in subcellular sites of ROS production (e.g. chloroplasts and mitochondria contributes to ROS scavenging in *Arabidopsis*. An intriguing link has been evidenced by Dave et al. (2011), who found that 12-oxo-phytodienoic acid (OPDA), a precursor of the oxylipin jasmonic acid, is accumulated in *Arabidopsis* mutants affected in peroxisomal β-oxidation and characterized by impaired seed germination. High OPDA levels correlated with inhibition of seed germination, suggesting for a role as signaling molecule. In addition, ROS trigger oxylipin accumulation and *cis*-OPDA contains a chemically reactive α,β-unsaturated carbonyl structure that binds to free thiol groups and modify cellular proteins (Dave and Graham 2012). Indeed, α-linolenic acid (18:3, ω-3) is oxidized by lipoxygenases into either 9- or 13-hydroperoxy-octadecatrienoic acids, which are metabolized into different oxylipins (Andreou and Feussner 2009), and allene oxide synthase is specifically implicated into the formation of jasmonic acid (Browse, 2009). In agreement with this, results hereby reported highlight a connection between the seed fatty cohort and oxylipins, mediated by α-linolenic acid (18:3, ω-3). In-depth analyses carried to better understand the interplay between ROS and oxylipins in the pre-germinative metabolism might open a new interesting molecular scenario related to seed vigor.

Djemel et al. (2005) reported a similar FA composition in dry seeds of *M. truncatula* Jemalong 5. The major components were C16:0, C18:1, C18:2, and C18:3, accounting for approximately 95% of the total FA content. Accordingly, in a different *M. truncatula* accessions, SA1619, the most abundant FAs were C16:0 (palmitic acid), C18:1 ω-9 (oleic acid), C18:2 ω-6 (linoleic acid), and C18:3 ω-3 (alpha-linolenic acid) (Song et al., 2017).

A schematic representation of the metabolic events occurring along the different pathways monitored in imbibed seeds is provided in **Figure 5**. The progressive increase in free radical species, ROS and RNS, observed throughout imbibition resulted in oxidative damage, triggering protective responses, according to temporally defined profiles. This is documented by the decrease in thiols, indicative of cellular redox dynamics. However, this picture becomes even more complex when considering the protective role played by NO against oxidative stress during seed germination (Simontacchi et al., 2004; Hu et al., 2007). Furthermore, ROS and RNS levels determine the balance between signaling events and oxidative stress (Bailly et al., 2008; Rajjou and Debeaujon, 2008; Arc et al., 2011; Rajjou et al., 2012). Fluctuations in lipid peroxidation and tocopherols are consistent with an active role of these antioxidants in membrane protection. Significant changes in the FA cohort appeared at the earliest step of imbibition (0.5 h) hereby investigated (**Figure 5**). The ω-3 DPA might be regarded as a very early indicator of the molecular events occurring at the level of cellular membranes, in terms of physical properties and signaling. Although no reports are currently available in plants, it is worth noting that in animal cells ω-3 DPA can influence the expression of genes encoding pro-inflammatory factors (Tian et al., 2017). At 8 h of imbibition, the observed peak in tocopherols and the additional changes in the FA cohort further boost the seed repair response (**Figure 5**). At this timepoint, the ω-3 DPA is still required to handle membranes whereas the occurrence of enhanced levels of ω-6 γ-linolenic acid could be regarded as another distinctive hallmark of the *M. truncatula* pre-germinative metabolism.

## Conclusions

This work looks into the pre-germinative metabolism of a legume seed from the perspective of the lipid component, catching the qualitative and quantitative dynamics of the FA cohort. The purpose was to gain knowledge on the events crucial for membrane stability and function occurring in seeds challenged with oxidative stress during rehydration under physiological conditions. Besides this, we wanted to assess the potential of the FA cohort as a source of indicators useful to monitor the progression of seed metabolism in relation to quality, in agreement with previous studies addressing the contribution of DNA repair and antioxidant genes, as well as metabolites. Among the list of FAs showing changes in their quantitative profile, the ω-3 DPA appears an attractive starting point for future investigations oriented towards some basic aspects of seed biology/technology. Is the ω-3 DPA an up-stream component of specific signaling pathways activated at the beginning of imbibition? Does it target genes involved in the pre-germinative metabolism and eventually affecting seedling vigor? Future work will be oriented towards finding a proper response to these research questions, analyzing either other legume species or seeds subjected to different type of environmental stress (e.g. drought).

## Data Availability Statement

Publicly available datasets were analyzed in this study. This data can be found here: https://doi.org/10.1111/pce.13342.

## Author Contributions

AB, SA, AM, and CF conceived the work and wrote the manuscript; ED, AP, AM, and AL contributed the experimental data; AB, SA, AM, CF, ED, and AP contributed to the discussion of data. All authors read and agreed with the final version of the manuscript.

## Funding

This work was supported by CARIPLO Foundation in the frame of the WAKE-APT project (Code 2016-0723) (“Seed Wake-up with Aptamers: a New Technology for Dormancy Release and Improved Seed Priming”) and by the Italian Ministry of Education, University and Research (MIUR): *Dipartimenti di Eccellenza* Program (2018–2022) - Dept. of Biology and Biotechnology “L. Spallanzani,” University of Pavia (to AP, AM, AB, ED). The financial support from *Fundação para a Ciência e a Tecnologia* (Lisbon, Portugal) is acknowledged through research unit GREEN-it “Bioresources for Sustainability” (UID/Multi/04551/2019) and SA PhD Holder Contract (DL57). Sponsorship from COST Action FA1306: “The quest for tolerant varieties: phenotyping at plant and cell level” is gratefully acknowledged. AP has been awarded by a PhD Fellowship from IUSS-*Scuola Universitaria Superiore Pavia*. CF and AB thanks the collaborative environment created by the EU COST Action CM1201 “Biomimetic Radical Chemistry.” The authors would like to thank Dr. Danny Alexander (Metabolon, US) for his support during the preparation of this manuscript.

## Conflict of Interest

The authors declare that the research was conducted in the absence of any commercial or financial relationships that could be construed as a potential conflict of interest.
