# Supplementary material for: How Does the Seed Pre-Germinative Metabolism Fight Against Imbibition Damage? Emerging Roles of Fatty Acid Cohort and Antioxidant Defence
Source: Front Plant Sci. 2019 Nov 21;10:1505. doi: 10.3389/fpls.2019.01505 (PMC6881478; doi:10.3389/fpls.2019.01505)
Supplement: Supplementary file 1 [file DataSheet_1.docx]

Supplementary Material

**How Does the Seed Pre-germinative Metabolism Fights against Imbibition Damage? Emerging Roles of Fatty Acid Cohort and Antioxidant Defence**

**Enrico Doria^1,#^, Andrea Pagano^1,#,⸹^, Carla Ferreri^2^, Anna Vita Larocca^3^, Anca Macovei^1^, Susana de Sousa Araújo^4^, Alma Balestrazzi^1,*^**

^1^ Department of Biology and Biotechnology 'L. Spallanzani', via Ferrata 9, 27100 Pavia, Italy,

^2^ Consiglio Nazionale delle Ricerche, Research Area of Bologna, Via P. Gobetti 101- 40129 Bologna (Italy)

^3^ Lipinutragen srl, Laboratorio di Lipidomica, Via P. Gobetti 101, 40129 Bologna

^4^ Instituto De Tecnologia Quìmica e Biologica ITQB-UNL António Xavier (ITQB-UNL) Avenida da Republica, Estação Agronómica Nacional 2780-157-Oeiras, Portugal

^#^ These authors equally contributed to the work

^⸹^ Present address: Institute of Plant Genetics, Polish Academy of Sciences, Poznan, Poland

*** Correspondence:**Alma Balestrazzi
[alma.balestrazzi@unipv.it](mailto:alma.balestrazzi@unipv.it)


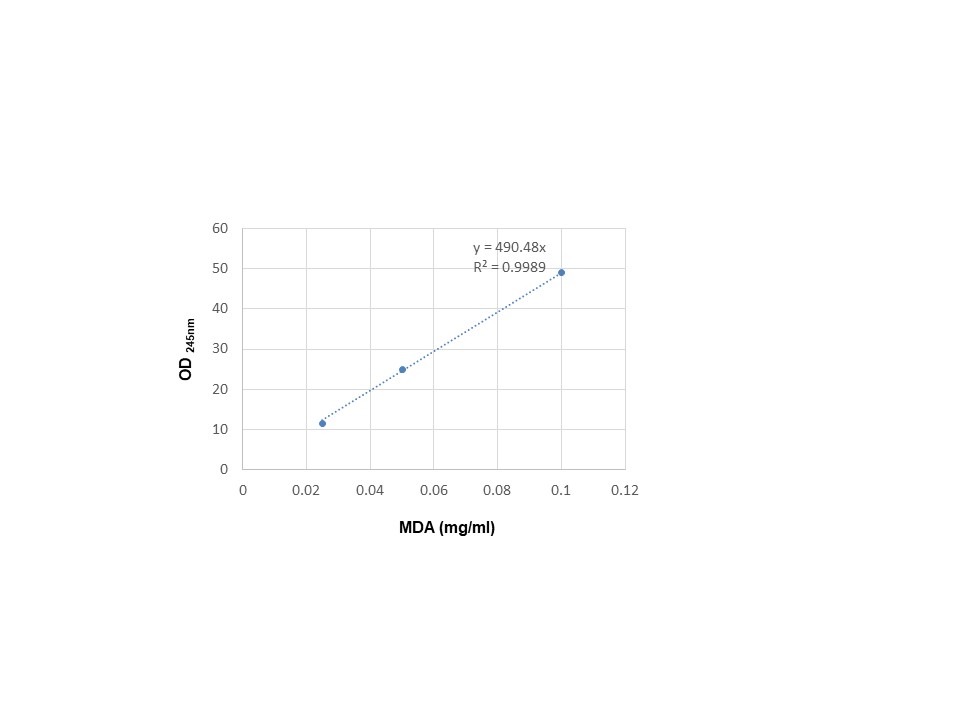


**Supplementary Figure S1**. MDA standard curve measured at 254 nm using an UV-visible spectrophotometer (UV-1800, Shimadzu, U.K.).


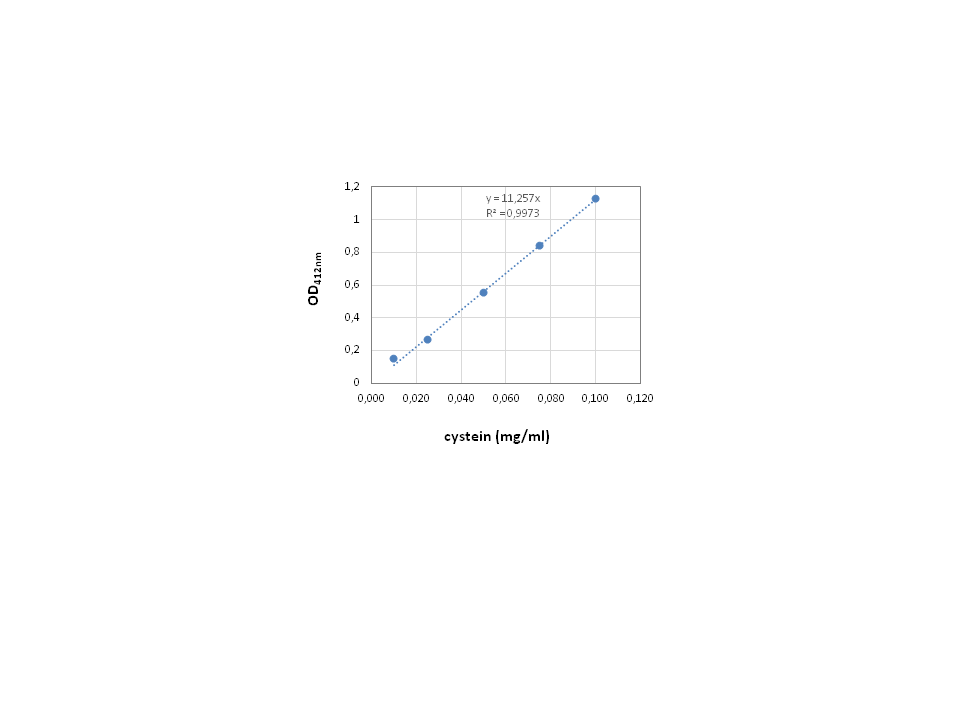


**Supplementary Figure S2**. Cysteine standard curve constructed using cysteine concentrations of in the range of 0.01-0.1 mg/ml and used for the quantification of the thiols measured spectrophotometrically at 412 nm.


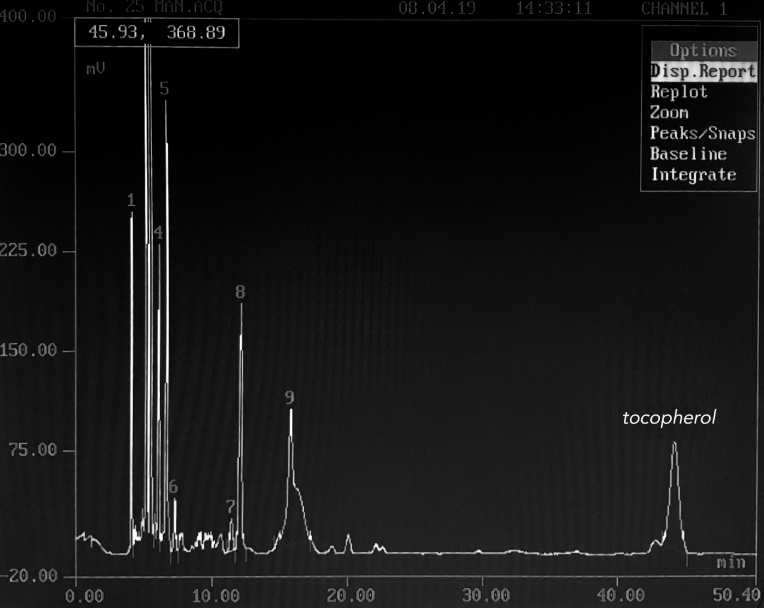


**Supplementary Figure S3**. Chromatogram showing the detection of γ-tocopherol by the HPLC system (Kontron Instrument 420 system) equipped with a C18 column (250 x 4.6 mm, 5μ).


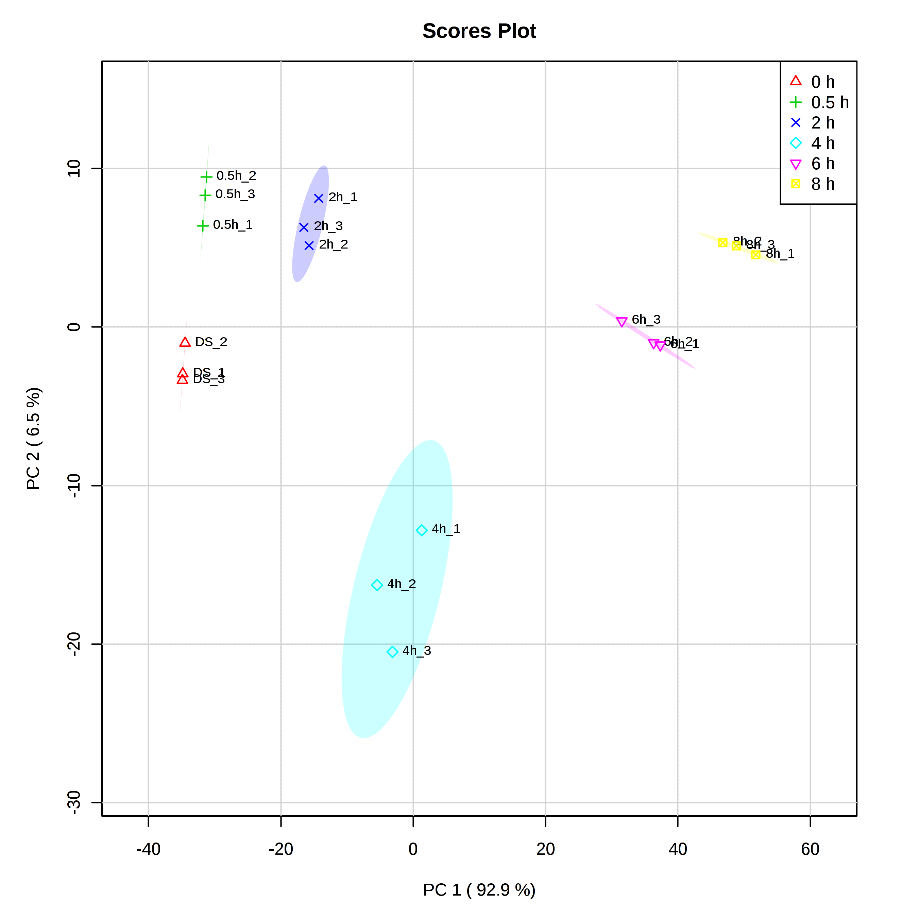


**Supplementary Figure S4.** Principal component analysis score plot explaining 99.3% of the variance of measured variables (ROS, RNS, MDA, thiols, tocopherols, SFA, MUFA, FUPA, ω-6, ω-3, and trans-fatty acids), in which PC1 accounts for 92.9%, whereas PC2 accounts for 6.5% of total variance.

**Supplementary Table S1.** Principal Component Analysis (PCA) loading scores corresponding to each principal component (PC) based on the used variables (ROS, RNS, MDA, thiols, tocopherols, SFA, MUFA, FUPA, ω-6, ω-3, and trans-fatty acids). The data were retrieved from MetaboAnalyst program available at <https://www.metaboanalyst.ca>.

**
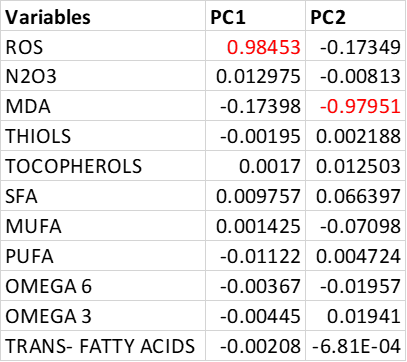
**

**Supplementary Table S2**. Principal Component Analysis (PCA) loading scores corresponding to each principal component (PC) based on the used variables (myristic acid, palmitic acid, stearic acid, arachidic acid, behenic acid, linolelaidic acid, linoleic acid, γ-linolenic acid, dihomo γ-linolenic acid, α-linolenic acid, sapienic acid, palmitoleic acid, oleic acid, vaccenic acid, docosopentaenoic acid, docosohexaenoic acid). The data were retrieved from MetaboAnalyst program available at <https://www.metaboanalyst.ca>.
